# Quality of Serious Illness Communication with Hospitalized Limited English Proficient Patients: A Mixed Methods Study

**DOI:** 10.1089/pmr.2025.0005

**Published:** 2025-05-22

**Authors:** Laura M. Holdsworth, Samantha M. R. Kling, Marcy Winget, Heather Z. Mui, Donn W. Garvert, Darlene Veruttipong, Briththa Seevaratnam, Sonia Harris, Winifred Teuteberg

**Affiliations:** ^1^ Division of Primary Care and Population Health, Department of Medicine, Stanford University School of Medicine, Palo Alto, California, USA.; ^2^ School of Social Work, University of Minnesota, St. Paul, Minnesota, USA.

**Keywords:** advance care planning, health communication, inpatients, qualitative research, Limited English proficiency

## Abstract

**Background:**

The Serious Illness Conversation Guide was developed to support high quality goals of care conversations with seriously ill patients; however, guide implementation for patients with limited English proficiency (LEP) has not been studied. This evaluation aimed to explore serious illness conversations with hospitalized LEP patients, defined as those with a non-English language documented, from clinician and interpreter perspectives; and assess differences in documentation in the electronic medical record (EMR) as a quality improvement effort.

**Methods:**

Parallel mixed methods evaluation including thematic analysis of observations and interviews with medical interpreters (*n* = 14), occupational therapists (*n* = 9), registered dietitians (*n* = 6), and resident physicians (*n* = 3) of a quaternary academic hospital in the United States. Comparison of EMR documentation for hospital admissions with English proficient (*N* = 7396) and LEP patients (*N* = 2326).

**Results:**

Six themes characterized serious illness communication and guide use with LEP patients. As compared to other clinical encounters, both interpreters and clinicians perceived serious illness communication as unique. Both groups acknowledged that interpreters convey meaning, though being an effective voice of the clinician required advanced preparation of the interpreter, even when the guide was used. There were no differences in documentation between the groups (4.7% (345/7396) versus 5.4% (126/2326); *p* = 0.21).

**Conclusions:**

Even when the guide is used, there may be differences in serious illness communication quality with LEP patients depending on how clinicians engage with the guide and interpreter preparation. The guide may be a method to enhance communication quality, but for LEP patients, requires the parallel implementation of workflows that support high-quality communication.

## Introduction

Serious illness communication is essential for patient-centered care,^1^ yet there are often barriers to timely, high-quality conversations.^2,3^ Most clinicians do not receive evidence-based communication skills training needed for high-quality communication with seriously ill patients.^4^ The Serious Illness Conversation Guide (SICG) was developed by Ariadne Labs (Boston, Massachusetts, USA) to support goals of care conversations with seriously ill patients.^5^ It provides a structured, scripted approach to elicit patients’ understanding about their health condition, share prognostic information, and explore goals and values to inform treatment recommendations. While there is some evidence supporting the effectiveness of the SICG,^6–9^ patients with limited English proficiency (LEP) have largely been excluded from these and other studies testing communication tools.^10^

Communication with LEP patients can differ in important ways to communication with English proficient patients: clinician communication may be modified and less frequent, clinician ability to assess patient and family understanding is impaired, and relationship building is more difficult.^11^ Additional cultural barriers, such as faith-based beliefs about treatment and family approaches to decision-making, can also hinder communication.^11^ Interpreters, therefore, play an important role in facilitating communication; not only do they translate language, but they can verify and support patient understanding, and transmit information that captures cultural nuance.^12^

Given the known complexities around communicating with LEP patients and that the SICG has required adaptation to suit different cultural contexts within English-proficient populations,^13,14^ this evaluation aimed to investigate use of the SICG for conversations with LEP patients in a hospital setting. Objectives included: (1) explore the occurrence of serious illness conversations with LEP patients, from both clinician and interpreter perspectives; and (2) assess differences in documentation of SICG conversations between English proficient and LEP patients.

## Methods

We conducted a mixed-methods evaluation in which qualitative and quantitative data were collected and analyzed in parallel; the results of each method informed the overall interpretation presented in this paper.^15^ The evaluation received a nonresearch determination from the Stanford Institutional Review Board as a quality improvement effort.

### Setting and population

This evaluation took place in a 605-bed, quaternary academic hospital with an affiliated National Cancer Institute-designated cancer center adjacent to the hospital in the United States. This evaluation focused on the in-hospital medicine and oncology units.

In the hospital, SICG implementation efforts used a team-based approach including physicians, occupational therapists (OTs), and/or registered dietitians (RDs); this team-based approach has been previously described.^16^ In the team model, only physicians discuss prognosis, but all trained clinicians can ask scripted questions about quality of life, values, goals, and make recommendations within their scope of practice. As the goal of the evaluation was to understand how the scripted SICG is used with LEP patients, we sampled across the three clinical groups to ensure we captured the experiences of all groups who use the guide in this setting. Patients are identified as suitable for SICG conversations using clinical judgement, which could be supported by an electronic medical record (EMR)-based flag that indicates 12-month mortality risk.^17^ For both observations and interviews, clinician participants were identified by clinical and administrative leaders and champions of the SICG who were involved in its implementation.

### Observations

We first conducted ethnographic participant observations with clinicians (RDs and OTs) and interpreters. For the first observation with each group, two researchers (LMH, HZM) observed together to ensure consistency in observational approach; subsequent observations were conducted by one researcher. Both researchers are trained in qualitative methods and are native English speakers; HZM also speaks Cantonese. We observed clinicians to understand their daily routine, how they identify seriously ill patients, barriers to conducting serious illness conversations, and interactions with interpreter services. Because interpreter services are on-demand without clinical interactions known in advance, observations with interpreters focused generally on their preparation for clinical encounters, and interactions with clinicians, patients, and families. Observations were scheduled in 2–3-hour blocks with the first 15–30 minutes spent getting to know the participant, discussing their daily schedule, and, for clinicians, discussing their patient lists. Observers asked questions between encounters to clarify actions and understand participant perspectives. Handwritten notes made during the observational visit, including any direct quotes, were transcribed into a narrative format for analysis immediately following observations.

### Interviews

Following the completion of observations, we conducted in-depth interviews with a different group of clinicians (RDs, OTs, and residents, who are licensed physicians, but not yet independent practitioners) and interpreters to further explore experiences and gain a wider range of perspectives. In-depth interviews were conducted independently by LMH or HZM. Interviews with clinicians covered the following topics: experiences in caring for LEP patients, experiences with interpreter services, how the SICG is used in practice, and challenges encountered during serious illness conversations, especially with LEP patients. Interviews with interpreters covered complementary topics: experiences of interpreting for seriously ill patients, challenges encountered when interpreting, interactions with clinicians and patients/families, and awareness of the SICG (see Supplementary Data). Interviews were conducted over Zoom, recorded with permission, and transcribed for analysis.

### Qualitative data analysis

We carried out a thematic analysis using a codebook to facilitate a deductive and inductive approach.^18,19^ After all data were collected and researchers had familiarized themselves with the data, researchers met to develop an initial codebook derived from the topic guide and themes that had been noted during data collection. Interview transcripts and observation notes were imported into NVivo for analysis. HZM coded all data, with a portion second-coded by LMH. New themes were added to the codebook during coding and discussed by the researchers. Coded data were reviewed by LMH for recurrent patterns and organized into themes.^20^

### EMR data and analysis

Patient demographic and clinical data, language, and updates to the SICG note from unplanned hospital admissions to medicine and oncology service lines between August 1, 2022 and July 31, 2023 were extracted from Epic EMR (Epic Systems, Verona, WI, USA). The SICG note is an institution-specific, EMR-based templated note designed for documentation of SICG conversations.

Means, standard deviations, and range were calculated for patient age, Charlson Comorbidity Index,^16,17^ and admissions. Frequencies and percentages were also calculated for the remaining patient characteristics and for characteristics of total admissions. Differences in characteristics between English proficient and LEP patients were calculated using two independent sample *t-test* and chi-square test. Mixed-effect logistic regression models were generated to calculate differences between English-proficient and LEP patients in the documentation of SICG notes during admissions. *P* values <0.05 were considered statistically significant. Analyses were performed using SAS (version 9.4; SAS Institute, Inc., Cary, NC, USA).

## Results

We carried out 10 observational visits with RDs, OTs, and interpreters totaling 22 hours between May and September 2023. All observations involved an LEP patient encounter, and/or use of the SICG. Twenty-two interviews with RDs, OTs, residents, and interpreters were conducted between September and November 2023. Interviews lasted on average 34 minutes (range 26–45). Qualitative data collected for each group are presented in Table 1. Four clinicians reported speaking languages other than English proficiently.

**Table 1.** Qualitative Data Collected by Method and Professional Group

Professional group	Observations (*n* = 10)	Interviews (*n* = 22)
Interpreters^a^	4	10
Registered dietitians	3	3
Occupational therapists	3	6
Residents	—	3

^a^ Interpreter working languages included: Spanish, Portuguese, Russian, American Sign Language, Mandarin, Cantonese, Farsi/Dari.

### Patient characteristics and SICG documentation in inpatient setting

There were statistical differences between English-proficient and LEP patients in most demographic characteristics (Table 2). LEP patients were older than English proficient patients (68.0 (17.1) vs. 62.6 (19.1) years; <0.01). A larger proportion of LEP patients identified as Hispanic or Latino or as Asian or Pacific Islander, and a higher proportion had Medicaid insurance. A larger proportion of LEP patients had no diagnostic history within the health care system compared to English-proficient patients (27.6% (413/1498) vs. 21.4% (1045/4878)). Utilization of the SICG notes during hospital admissions did not differ between LEP and English proficient patients (5.4% (126/2326) vs. 4.7% (345/7369); *p* = 0.21; Table 2).

**Table 2.** Characteristics of Patients by Language Proficiency

Patient characteristics				
Characteristic	All patients	English proficient	LEP^a^	*P* value^b^
Total, *N* (%)	6376 (100%)	4878 (76.5%)	1498 (23.5%)	
Age				<0.01^c^
Mean (SD)	63.9 (18.8)	62.6 (19.1)	68.0 (17.1)	
Range	17.9–104.1	17.9–104.1	19.5–101.7	
Sex, *n* (%)				<0.01^d^
Female	3251 (51.0%)	2450 (50.2%)	801 (53.5%)	
Male	3125 (49.0%)	2428 (49.8%)	697 (46.5%)	
Race/ethnicity, *n* (%)				<0.01^d^
White	2668 (41.8%)	2595 (53.2%)	73 (4.9%)	
Hispanic or Latino	1504 (23.6%)	776 (15.9%)	728 (48.6%)	
Asian and Pacific Islander	1367 (21.4%)	781 (16.0%)	586 (39.1%)	
African American or black	363 (5.7%)	359 (7.4%)	4 (0.3%)	
Other	401 (6.3%)	305 (6.3%)	96 (6.4%)	
Unknown/declined to state	73 (1.1%)	62 (1.3%)	11 (0.7%)	
Charlson Comorbidity Index Risk Score^e^				0.07^f^
*N*	4918	3833	1085	
Mean (SD)	3.1 (3.2)	3.0 (3.2)	3.2 (3.1)	
Range	0.0–19.0	0.0–16.0	0.0–19.0	
Charlson Comorbidity Index Risk Group,^g^ *n* (%)				<0.01^d^
No Charlson comorbidities (risk score: 0)	1278 (20.0%)	1026 (21.0%)	252 (16.8%)	
Low risk (risk score: 1–2)	1600 (25.1%)	1249 (25.6%)	351 (23.4%)	
Intermediate risk (risk score: 3–4)	764 (12.0%)	581 (11.9%)	183 (12.2%)	
High risk (risk score: 5 or more)	1276 (20.0%)	977 (20.0%)	299 (20.0%)	
No diagnosis information	1458 (22.9%)	1045 (21.4%)	413 (27.6%)	
Patient insurance, *n* (%)				<0.01^d^
Private	1371 (21.5%)	1280 (26.2%)	91 (6.1%)	
Medicare	3350 (52.5%)	2589 (53.1%)	761 (50.8%)	
Medicaid	1528 (24.0%)	899 (18.4%)	629 (42.0%)	
Other	69 (1.1%)	64 (1.3%)	5 (0.3%)	
Missing	58 (0.9%)	46 (0.9%)	12 (0.8%)	

Admissions characteristics				
	All admissions	Admissions with English proficient patients	Admissions with LEP patients	*P* value^h^
Total number of admissions (%)	9722 (100%)	7396 (76.1%)	2326 (23.9%)	—
Unplanned admissions per patient^i^				
Mean (SD)	1.5 (1.2)	1.5 (1.2)	1.6 (1.1)	0.27^c^
Range	1.0–16.0	1.0–16.0	1.0–16.0	
Service line, *n* (%)				
Medicine	6195 (63.7%)	4776 (64.6%)	1419 (61.0%)	0.07^j^
Oncology	3527 (36.3%)	2620 (35.4%)	907 (39.0%)	
SICG notes updated, *n* (%)	471 (4.8%)	345 (4.7%)	126 (5.4%)	0.21^j^

^a^ Patients who indicated a non-English language were categorized as having LEP.

^b^ *P* value from statistical tests assessing differences between patients with English proficiency and those with LEP.

^c^ *P* value from two independent sample *t-test* Satterthwaite.

^d^ *P* value from Chi-square test.

^e^ Charlson Comorbidity index was not calculated for patients who had no diagnostic history at the health care system.

^f^ *P* value from two independent sample *t-test* pooled.

^g^ Charlson Comorbidity Index was calculated from ICD-10 codes from one year prior to the patient’s first hospital admission. Scores had a range from 0 to 29 with higher scores indicative of poorer health.^20,21^

^h^ *P* value from statistical tests comparing admissions with patients with English proficiency and admissions with patients with LEP.

^i^ Unplanned admissions are defined as urgent and emergent admissions.

^j^ *P* value from mixed effects logistic regression models adjusted for random effects of patient and Charlson Comorbidity severity.

LEP, Limited English Proficiency; SICG, Serious Illness Conversation Guide.

### How the SICG has been implemented for hospitalized patients

All clinicians had received training in using the SICG and documenting conversations using a structured, templated note (i.e., SICG note). Only one resident who had a rotation in palliative medicine had additional serious illness communication training beyond SICG training. Clinicians described adapting the script to suit their communication style. Many clinicians did not use the full scripted language for all conversations, but rather would use one or two questions as needed. Only one clinician was observed to have referenced the physical guide while having a conversation. Most participants reported using the templated note for documenting conversations (i.e., SICG note) only if they intended to have a SICG conversation or, in a spontaneous conversation, asked the questions in a format similar to the guide.

### Clinician and interpreter perspectives of serious illness conversations with LEP patients

Six themes characterized serious illness communication with LEP patients from the perspectives of clinicians and interpreters; exemplar quotes are presented in Table 3.

**Table 3.** Themes with Example Quotes

Theme	Example quotes
Serious illness communication is a special case	“A person that is an English-speaking person I may go one or two sessions. When it’s a Latino, it may take me four sessions or more to be able to get that rapport, understanding, knowing what they don’t know and what they’re going to need help with.” (Occupational Therapist 10)
	“I remember the doctor said, ‘You are dying and that’s why we sent you to the-’ I don’t remember if it was a nursing home or hospice. ‘You need to accept that so you don’t have to come back because we already talked about this.’ So I remember her eyes were just touched in and so it was very ... Finally, she accepted at that moment. And unfortunately for me, I had to say it. I said everything the doctor’s saying. I have to repeat. And she looked at me, she said, ‘So I’m dying.’ I said, ‘Yes.’ So yeah, it was very sad.” (Interpreter 02)
Establishing rapport differently (clinician only)	“I think it’s different in that the process of establishing rapport looks different. There might need to be a bit more effort on part of the provider to establish that connection that you would with someone who you speak the same language. So eye contact. Really ensuring that I’m looking at the patient as opposed to at my phone or at the interpreter if they’re physically present.” (Occupational Therapist 05)
The voice of the clinician (interpreter only)	“The pattern of the discourse is really important, and that’s where being prepared really helps. […] For instance, in cancer treatments, the doctors will start using all the chemical compound names. You don’t even know what they’re saying, and you trip up over that, and you lose confidence. […] Pretty soon you just get mentally very, very tired, and you’re shaky. It’s not a good look. It’s not a good look when we’re delivering bad news.” (Interpreter 09)
	“If the conversation is not going well, there’s very little that the interpreter can do within our code of ethics to compensate. […] I’ve heard another doctor who did it better, but I can’t substitute what he said for what you’re saying, because it’s not interpreting. That’s not ethically what I am here to do.” (Interpreter 03)
Interpreters help convey meaning	“I think there’s a nuance that the interpreter provides in the communication because it’s also not just the words but the emotion behind those or the beliefs of the patient. And I think it obviously adds a little bit of interpretation on the part of the interpreter, but I feel like I always appreciate when there’s nuance to the wording of things.” (Resident 01)
	“Sometimes the patients don’t know quite what I’m asking [in relation to critical abilities] or the response. For example, I’ve had a patient, all of [their critical abilities] were related to walking because walking was really important to them. But I think it wasn’t quite always what we were trying to get at with the question, but I didn’t necessarily want to prompt them with examples if that wasn’t really coming from the patient.” (Occupational Therapist 12)
	“In Latin America, and at least in my home country, most of the decisions are always made by the doctor and there’s not as much involvement of the patients. And so, when you have a patient that maybe for the first times or the first couple of times in their life, they’re being faced with very important decisions, life-changing decisions, they may feel very confused as to ‘why am I being asked if I’m not the expert?’ […] So a couple of times I’ve addressed the situation with the provider ahead of time and I’ve asked their permission if I could at least make a brief introduction. ‘Right now the provider’s going to explain this procedure so at the end, you can make a choice about what you want.’ And so that at least sets their mind into listening mode as opposed to ‘why are they telling me all of this?’” (Interpreter 06)
Difference in quality between in-person and telephone interpretation	“I really like having the in-person interpreter because I just feel like there’s no confusion [in what is being asked]. In addition, with the in-person, I find it’s more personable. This is a serious conversation, and then you’re telling it to someone on the phone that you can’t see. So [in-person] just feels more warm, I feel like. And then if the patient is hard of hearing, the interpreter can really get close to them.” (Occupational Therapist 08)
	“In person, it’s infinitely preferable. You have that moment. You overhear stuff. Maybe you’ve seen the nurse and you’ve been there for other encounters, because it isn’t just... Sometimes it’s pharmacy, sometimes it’s the nurses, sometimes it’s OT, speech pathology have come in, and so you may have seen the case from different dimensions. But you turn on the video, [the clinicians] don’t even think you need to know [about the case]. [...]And a lot of times you can’t even see what’s going on in the room, and they don’t think you need to see it. You have no idea.” (Interpreter 09)
Lack of standard practices for effectively engaging with interpreters	“I think particularly for the conversations like these more serious illness-guided discussions, [meeting beforehand] saves a lot of time, I think, during the interpretation. I think it actually allows for probably even better interpretation because the interpreter knows what’s anticipated. Whereas, I go into the conversation knowing what I’m going to say and how I’m going to guide it, giving them that ability too, and also to get a sense of the language they might anticipate from the patients.” (Resident 02)
	“In theory, our protocol states that before going into a room, the provider should give us some context, but sometimes, if not, most of the time, that doesn’t necessarily happen. Say for rounds, they’ll just say 'hi' to me and then we’ll walk right in, and that’s okay. We’re trained to work with that as well. But specifically for these type of [serious illness] encounters, I do find that that is not just helpful, it is essential. I need to be aware of what you’re going to say, and I need to be aware of what is already happening. Where are we? What does she know? Or what does he know? What is the goal of this conversation we’re about to have? What’s the tone I need to make sure I am following?” (Interpreter 04)

#### Serious illness communication is a special case

Serious illness conversations, regardless of whether they followed the SICG structure or were unstructured, were noted as unique because they are all different and difficult. All clinicians recognized that communicating with LEP patients and families took longer and added “a layer of complexity” (OT 24 observation) to serious illness conversations and care, in general, due to linguistic and cultural differences.

When asked about whether they had interpreted for any serious illness conversations, most interpreters recounted a specific patient story. These experiences were wide-ranging and not limited to chronic serious illness that the SICG is typically used for, but also included unexpected or traumatic deaths. In all cases, interpreters remembered these events in detail, suggesting that interpreting these conversations were formative, and possibly even traumatic, experiences.

#### Establishing rapport differently (clinician only)

Clinicians described needing to use simple, direct phrasing, or nonverbal techniques to establish rapport with LEP patients. Some clinicians reflected that rapport building was difficult not just because of linguistic differences, but also cultural differences.

#### The voice of the clinician (interpreter only)

Interpreters described a tension between their role as a conduit for the clinician and being the individual who directly delivers bad news to patients, often for the first time. Interpreters described that it could be difficult for them to convey the clinician’s tone and meaning when they were unprepared for difficult, emotive conversations. Interpreters said that poor communication was often because clinicians were speaking abstractly or choosing words that did not translate well into the patient’s language, but that the interpreter’s code of ethics prohibited them from changing words to help the clinician sound better in these situations.

#### Interpreters help convey meaning

Clinicians recognized that it was helpful when interpreters could express nuance or help the clinician express themselves to the patient. Interpreters described trying to form a cultural bridge between US medical practice norms and the patient’s cultural norms. Interpreters described that their understanding of the patient’s culture ensured an accurate exchange of information for clinical decision-making.

Interpreters perceived that sometimes patients did not understand what clinicians were telling them. They attributed these difficulties to providers using abstract language, euphemisms, or phrases that did not translate well. These varied by language, but some examples included “hospice,” “slippery slope,” “I’m afraid that …,” and “shifting toward therapies that are palliative in nature.” In the SICG, the phrase “critical abilities” was noted to precipitate some confusion when asked in English or interpreted in other languages.

#### Difference in quality between in-person and telephone interpretation

Clinicians generally recognized in-person hospital interpreters were better than telephone interpreters for serious illness conversations because of the gravity of the conversation. However, some clinicians preferred telephone interpretation, which was typically provided by a third-party company, because it was easier to get interpreters when needed.

Interpreters described that phone interpretation was harder than in-person, especially for difficult conversations, because it could be difficult to tell who is talking. Interpreters use nonverbal cues to help them express the right tone and identify the best words. Additionally, an opportunity to discuss the case with the clinician prior to the encounter was typically not an option for telephone or video interpretation.

#### Lack of standard practices for effectively engaging with interpreters

Residents received some training in how to work with interpreters during medical school, but RDs and OTs reported no formal training. Clinicians typically relied on trial-and-error or their own personal experiences of learning a language to guide their interactions with interpreters. For SICG conversations, some clinicians reflected that meeting with interpreters before the encounter led to better conversations because, in doing so, the clinician and interpreter prepared themselves for the language and tone of the conversation.

Interpreters confirmed that there was variability between clinicians as to whether they prepared them in advance or debriefed with them following the clinical encounter. Several interpreters noted that palliative care providers were an exception; this group of providers typically took time before and after clinical encounters to discuss with the interpreter, and used direct, clear language during the encounter.

## Discussion

To our knowledge, this is the first evaluation exploring the use of the SICG as a tool for conducting serious illness conversations among LEP patients. We identified that a few clinicians used the SICG in its entirety as designed, but rather relied on the training and selected phrasing when having serious illness conversations. Based on the EMR data, SICG conversations were documented at a similar proportion regardless of English language proficiency; in spite of the increased difficulty of having conversations through an interpreter. There may, however, be important differences in the quality of the conversations based on language. As the interpreter code of ethics requires translation of the words that are spoken, the interpretation of SICG questions may vary by encounter, especially as the quality of the conversation can depend on nonverbal cues that the interpreter may or may not have available (i.e., in-person vs. telephone interpretation), and whether the clinician has briefed the interpreter beforehand. These workflow issues, which are fairly standard across the field of medical interpretation,^21^ likely led to the challenges for serious illness communication that both interpreters and clinicians described. These challenges led clinicians to reflect that conversations with LEP patients were lower quality, which is problematic as high-quality, complex advance care planning interventions, such as the SICG, may “increase compliance with patients’ end of life wishes and satisfaction with care,”^22^ p. 1020).

Issues with verbatim interpretation for serious illness communication have also been noted in the literature.^12^ Interpreters in our evaluation described that often in serious illness communication, the words that clinicians choose do not convey the same meaning when translated verbatim, often leading to patient confusion and misunderstandings. Interpreters sometimes described trying to find words that would best convey the clinician’s meaning, though a lack of knowledge of the patient’s condition or treatment could lead to inaccurate interpretations. Interpreters in our evaluation emphasized many of the same points that have been identified as best practice when working with interpreters for clinical care: clinicians should brief interpreters about the goal or purpose of the encounter beforehand, arrange in-person interpreters when possible, use plain, direct language, and pause during conversations to allow for translation.^11,21,23,24^ While there has been work done to translate the SICG into other languages (e.g., https://www.vch.ca/en/rpace-translations), interpreters at this institution said that translated copies of the guide are not likely to be utilized due to both their code of ethics and workflow challenges. Translated guides may help interpreters prepare for clinical encounters by priming them with the words needed to convey the meaning, but it does not replace the need for improved workflows and pre-visit communication with the interpreter.

Our observational work identified that the interpreter’s relationship to the clinicians, patients, and families, and their integration into the clinical teams’ workflow likely influenced the quality of the clinical encounter. This was particularly notable in clinician perceptions of the superiority of in-person interpreters as they were perceived as being better able to convey and pick up on nuance, and some clinicians felt that interpreters acted as a cultural support for patients. This observation is contrasted with some interpreter perspectives in this and other studies that they are merely a conduit for communication.^25^ Medical interpretation is a skilled profession, and accurate interpretation is essential for informed decision-making. A previous study identified that patients may be more likely to complete an advance directive when a professional interpreter is used versus a family interpreter indicating the influential role they have in conveying clinical messages.^26^ Our results reiterate previous findings that interpreters are not invisible in clinical care, but rather have an important role in shaping serious illness communication between language discordant patients and clinicians.^27,28^

### Limitations

The evaluation is limited to a single medical center. In order to accommodate clinical priorities, observations with clinicians were limited to two- to three-hour sessions. Clinicians typically tried to identify one or two patients to demonstrate their process of using the SICG which we recognized was likely not typical for their workflow. We acknowledge that the multidisciplinary team-based approach to using the SICG may be unique; however, we perceive that the experiences of using the SICG with LEP patients are not profession dependent as all clinician groups can use the majority of the scripted guide and the questions asked are the same regardless of clinical expertise. Only conversations documented on the SICG note in the EMR were included in our analysis; we did not evaluate inequities in serious illness conversations documented elsewhere in the EMR.

### Conclusions and implications for practice

This evaluation found no differences in the documentation of SICG notes between admissions with English proficient and LEP patients. However, we identified important differences in how the SICG has been implemented for LEP patients, namely that the quality of conversations may vary depending on how clinicians engage with the guide and interpreter preparation. Translations of the SICG into other languages were not used during conversations, but may help interpreters to be better prepared going into an encounter, if they are aware of the purpose of the encounter in advance. The SICG may be a method to enhance the quality of serious illness communication, but for LEP patients, it requires the parallel implementation of workflows that are known to improve communication with LEP patients.

## Abbreviations Used

EMR: electronic medical record
LEP: limited English proficiency
OT: occupational therapist
RD: registered dietitian
SICG: Serious Illness Communication Guide
