# Supplementary material for: Quality of Serious Illness Communication with Hospitalized Limited English Proficient Patients: A Mixed Methods Study
Source: Palliat Med Rep. 2025 May 22;6(1):282–90. doi: 10.1089/pmr.2025.0005 (PMC12410327; doi:10.1089/pmr.2025.0005)
Supplement: Supplementary Data [file pmr.2025.0005_supplementarydata.docx]

**Topic guide - Interpreters**

| Main question | Probes |
| --- | --- |
| Introduction: I first want to get some background information about yourself. | |
| Can you tell me about your role at [hospital]? | - Working language(s) - FTE - Locations; hybrid, remote-only, in-person only |
| What do you like best about your job? |  |
| Thanks for that information. My next questions focus specifically on your experiences of interpreting for patients who are seriously ill in the hospital and who are having advance care planning conversations or discussions about their goals for care. Some examples of these types of conversations might include telling a patient that they have a life limiting illness or asking what types of care they want or don’t want if they were to get sicker. | |
| Can you think of any times when you have had to interpret for these types of conversations? (Give other examples if necessary, e.g. resuscitation, palliative care, hospice)  If YES: Can you tell me about it? | - Did you know what was going to be discussed beforehand? (if so, how did you get this information?) - How does interpreting for these types of conversations compare to others? - (If these conversations are more challenging): Is there anything that could be done to make interpreting for these conversations easier? |
| If NO: Are there any types of conversations that you find particularly difficult to interpret for? | - What makes it difficult? - Are there any kinds of emotionally difficult conversations? What makes them difficult? |
| Are there any phrases or terms that you find are particularly difficult to translate in the languages you work in? | - What do you do when you come across a phrase or term that doesn’t translate well? - Is there any feedback process with clinical teams to make sure you have accurately conveyed a concept in these situations where translation is difficult? - Do you ever provide feedback to clinicians about their English phrasing that makes it more or less difficult for you to convey their meaning to patients? |
| SICG: For some goals of care conversations there is a structured conversation guide that has been developed and tested. It helps clinicians to have these types of difficult conversations with patients by giving them scripted language they can use to elicit important information. (Screen share guide in English). The guide has been translated into 17 languages for use at [hospital]. Not all conversations at [hospital] follow this format, but some dietitians, occupational therapists, and residents are using the guide and others may be adopting it as well. | |
| Are you aware of the guide? | - If YES: have you used it before? If so, how did you use it? |
| Thinking about your workflow and how you interpret, would it be helpful to have a copy of the translated guide with you if you knew that the clinician wanted to have this specific type of conversation with the patient? | - Why would it/would not be helpful? - Is it feasible to have something like this with you/ what would be the challenges in having the guide? |
| Is there something else you’d like to add that I haven’t asked about? |  |

**Topic guide – clinicians**

| Main question | Probes |
| --- | --- |
| Introduction: I first want to get some background information about yourself. | |
| Can you tell me about your role at [hospital]? | - What units you work on - FTE - Types of patients cared for - Do you speak any languages other than English with your patients? What’s your proficiency level and QBS qualification? |
| What do you like best about your job? |  |
| Language: Thanks for that information. I now want to talk about caring for patients with Limited English Proficiency (LEP). | |
| In what ways is caring for patients with limited English proficiency different to caring for patients who prefer English? | - What are some of the main challenges in caring for LEP patients? - Is there anything in your practice that is harder to do with LEP patients than English speaking? - Are you aware of any hospital policies around caring for LEP patients? |
| What’s your experience of using interpreter services? | - How do you decide to use an interpreter? - What is your preferred way of using interpreter services? (in-person, video, or phone) Why? - Did you receive training in how to work with interpreters for clinical care? Where did you receive the training (e.g. residency, staff onboarding)? |
| SICG: I now want to talk about if and how you have serious illness conversations, and I’m specifically interested SICG structured conversations using the serious illness guide. | |
| Can you tell me about what training you have had in using the guide? | - When did you have the training at [hospital]? - Any other serious illness communication training? (e.g. Vital Talk) |
| Can you tell me about how you use SICG in practice? | - How often do you have SICG conversations? - What do you use as cues to whether a patient should have a conversation? - How do you select or prioritize patients for conversations? - What are the challenges in using SICG in practice? |
| In what ways is having SICG conversations with patients in English different to having them using an interpreter? | - Is language a barrier to having SICG conversations? In what way, or why not? |
| Is there something else you’d like to share about caring for LEP patients or SICG conversations that I haven’t already asked about? |  |
